# Cocaine-Induced Ventilation/Perfusion Mismatch Mimicking Pulmonary Embolism

**DOI:** 10.1177/2324709620906962

**Published:** 2020-02-13

**Authors:** Phani Keerthi Surapaneni, Temidayo Abe, Norberto Fas

**Affiliations:** 1 Morehouse School of Medicine, Atlanta, GA, USA

**Keywords:** cocaine, pulmonary embolism, V/Q scan

## Abstract

Pulmonary complications from cocaine use can range from bronchospasm to vasospasm leading to pulmonary infarction. Profound vasospasm may also lead to perfusion defects presenting as pulmonary embolism on ventilation-perfusion scan. A 65-year-old patient with a past medical history of substance abuse and chronic kidney disease presents to the emergency department with sudden-onset chest pain and shortness of breath. Ventilation-perfusion scan revealed filling defect most notably in the lingual lobe. He was later discharged on warfarin for the management of pulmonary embolism. The patient presented to the emergency department 2 weeks later with similar complaints; the international normalized ratio was subtherapeutic, and urine drug screen was positive for cocaine. Repeat ventilation-perfusion scan revealed no filling defects. Follow-up bilateral venous Doppler of lower extremities and D-dimer were within normal limits.

## Introduction

Cocaine is a widely used abuse stimulant with approximately 2.2 million users in the United States.^1^ Additionally, it is responsible for 31% of emergency department visits related to substance abuse.^2^ Route of administration is most commonly through snorting, intranasal, and intravenous. Once absorbed, it is rapidly taken up by different organs leading to varieties of complications through different mechanisms.^3^

Pulmonary complications are varied depending on the route, dose, and frequency of administration.^4^ It can range from severe bronchospasm when inhaled to bronchiectasis from chronic usage.^4^ In fact, cocaine is responsible for up to 30% emergency department visits related to asthma attacks in young adults.^5^ While its effects on the pulmonary vasculature are poorly understood, chronic usage has been linked to pulmonary hypertension, and severe vasospasm can lead to pulmonary infarction.^6^ We present a case of cocaine-induced ventilation-perfusion (V/Q) mismatch mimicking pulmonary embolism (PE).

## Case Report

**Figure 1.**
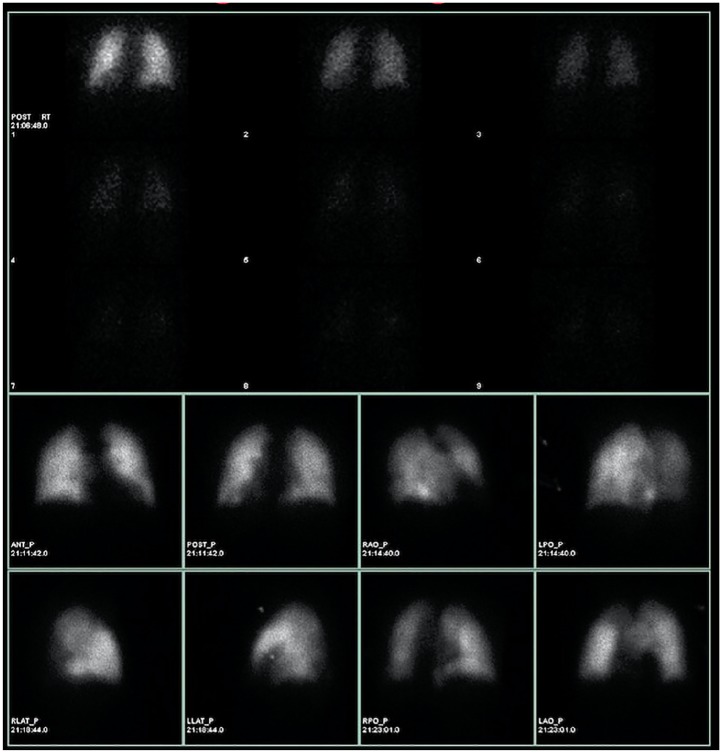
Ventilation-perfusion scan on initial admission. Top: ventilation. Bottom: perfusion. Note perfusion defects most pronounced in the left lateral region.

**Figure 2.**
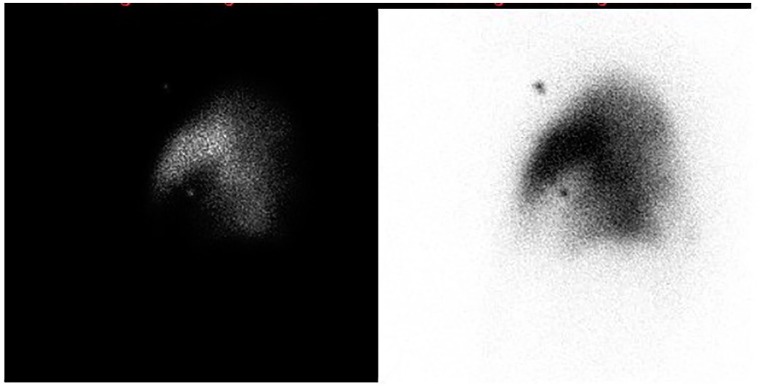
Dedicated perfusion images on the left lateral region with/without color scheme displaying perfusion defect.

A 65-year-old male with a past medical history of hypertension, diabetes, renal cell carcinoma, status-post nephrectomy, and chronic kidney disease stage IV presents to the emergency department with sudden chest pain and shortness of breath. He denies any fever, chills, or prior episodes. Social history was significant for a 20 pack-year smoking history and intravenous drug abuse. Vitals were blood pressure 160/95 mm Hg, pulse 90 beats per minute, respiratory rate 18 breaths per minute, and temperature 37.8°C. Physical examination was significant for a nonobese male in acute distress and chest wall tenderness on palpation. A 12-lead electrocardiogram obtained revealed no ischemic findings. Laboratory findings revealed a creatinine of 4.3 mg/dL, baseline (3.0-4.5 mg/dL). Other laboratory findings including troponin and brain natriuretic peptide were normal. Planar V/Q scan obtained 8 hours later revealed a large filling defect in the inferior lingula and small defects in the posterior inferior left lower lobe and posterior inferior right upper lobe characteristic for PE (Figures 1 and 2).

Follow-up transthoracic echocardiogram revealed no evidence of ventricular strain. The patient was started on heparin drip and admitted for further management.

He remained stable throughout the admission and was successfully bridged to warfarin. He was later discharged home in stable condition, and international normalized ratio at that time was 2.3.

**Figure 3.**
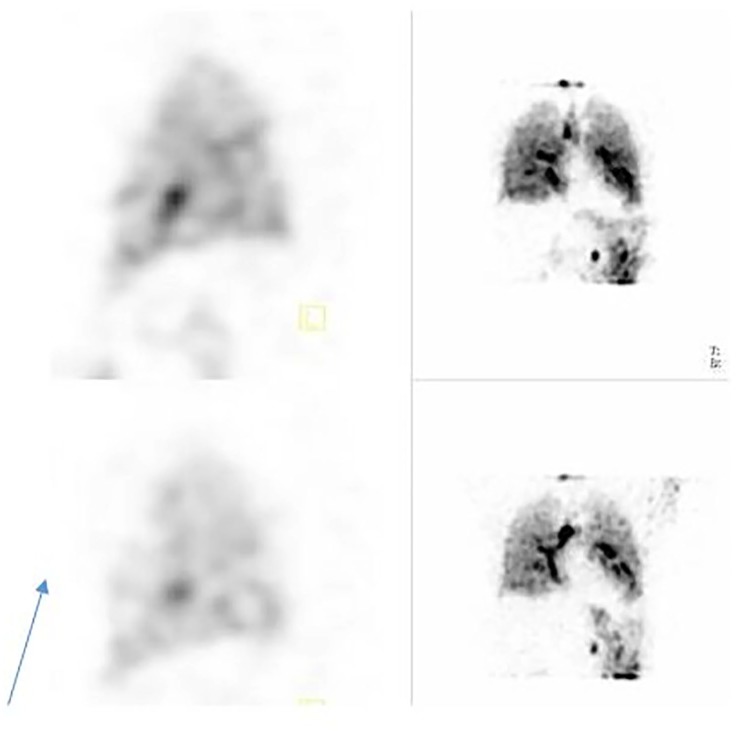
SPECT (single-photon emission computed tomography) ventilation-perfusion on readmission sagittal view. Top: ventilation images. Bottom: perfusion images. Note no perfusion defects previously seen.

**Figure 4.**
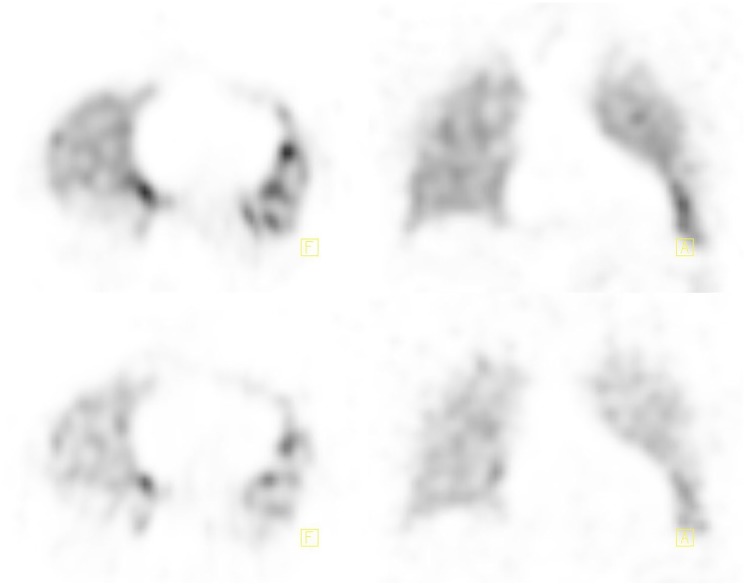
SPECT (single-photon emission computed tomography) ventilation-perfusion on readmission axial and coronal view. Top: ventilation images. Bottom: perfusion images. Note no perfusion defects previously seen.

Two weeks later, he presented to the emergency department with similar complaints. Vitals were unremarkable, and physical examination was unchanged from prior. Significant laboratory finding was the international normalized ratio of 1.12. The patient reports nonadherence to warfarin. Due to concern for recurrent PE, 2 hours into admission, we obtained repeat V/Q scan, this time with single-photon emission computed tomography (SPECT) owing to improved sensitivity and specificity as well as its enhanced ability to differentiate resolving from acute perfusion defects. It revealed no filling defects (Figures 3 and 4).

Lower extremity Doppler ultrasound revealed no clot, and D-dimers were normal. Urine drug screen obtained was positive for cocaine. He later admitted to inhaled cocaine use a few hours prior to presentation. With the help of the radiologist, we compared both V/Q scans, and it was deemed that the filling defect initially identified was due to vasospasm of the pulmonary vessels, likely secondary to cocaine.

Warfarin was discontinued, cocaine cessation was advised, and his chest pain was managed as musculoskeletal pain. He was discharged home in stable condition.

## Discussion

Cocaine is a widely used abuse stimulant with approximately 2.2 million users in the United States.^1^ Additionally, it is responsible for 31% of emergency department visits related to substance abuse.^2^ Route of administration is varied, and its half-life is about 60 to 90 minutes; once absorbed, it is rapidly taken up by different organs leading to varieties of complication through different mechanisms.^3^

The cardiovascular effects and complications of cocaine have been extensively studied, and they are thought to be secondary to various pathogenic mechanisms. First, one of its active metabolite benzoylmethylecgonine inhibits epinephrine and norepinephrine reuptake at neural synapses, which potentiates endogenous catecholamines release, resulting in profound hypertension, tachycardia, and such increased arterial wall shear stress.^7-9^ This can result in the separation of the tunica media from the intima leading to aortic or coronary artery dissection even in those without underlying risk factors.^7,8^ Second, intense vasoconstriction from catecholamine surge, increased endothelin, and decreased nitric oxide may lead to coronary vasospasm, myocardial oxygen supply-demand mismatch manifesting as angina, or myocardial infarction. Last, endothelial damage from shear stress as explained above, coupled with increased fibrinogen and von Willebrand factor, promote platelet aggregation and thrombi formation. When the coronary vessels are involved, myocardial infarction ensues, in the cerebral vessels, hemorrhagic or ischemic stroke and PE in the pulmonary vessels.^9-11^

Few data exist on the effects of cocaine on the pulmonary vasculature. It has been associated with pulmonary hypertension, infarction, and thromboembolism. They are thought to be secondary to the cocaine-induced vasoconstriction or vasospasm, granulomatous process, and endothelial dysfunction.^4,6,12,13^ Furthermore, intravenously administered cocaine can lead to foreign body (needle fragments) emboli causing PE or aseptic pulmonary granuloma.^14,15^ In our case, we believe that the filling defect initially detected on the V/Q scan was related to cocaine abuse.

The V/Q scan is an imaging modality used in the evaluation of PE.^16^ It is relatively safe and often used when computed tomography pulmonary angiography is contraindicated. Each lung segment is supplied by a single-end pulmonary artery. Adequate gas exchange requires a balance between alveolar ventilation and pulmonary blood flow in that lung segment.^17^ In-balance between alveolar ventilation and perfusion is termed V/Q mismatch. The V/Q scan relies on this principle to identify medical conditions that can lead to V/Q mismatch via ventilation defects or perfusion defects.^17^ In the case of PE, occluded pulmonary vessels prevent adequate blood flow to well-ventilated segments of the lungs leading to perfusion defects. PE is reported if there is V/Q mismatch in at least 1 lung segment or 2 subsegments that follows a single pulmonary artery.^17^ A normal scan can safely exclude PE with a negative predictive value close to 99%, while the probability of having PE with a high probability scan is approximately 96%.^16^ Despite its excellent diagnostic utility, it has some pitfalls. It is not 100% specific for PE as any cause of pulmonary artery occlusion will present as perfusion defects on V/Q scan. Examples include vasculitis, neoplastic obstruction of the pulmonary vessels, granulomatous disease affecting the pulmonary vessels, and pulmonary vein stenosis.^18-21^

In this case, we believe that severe vasospasm from cocaine inhalation was responsible for the perfusion defect noted on the initial V/Q scan. To our knowledge, only 2 cases of cocaine-induced perfusion defects have been reported in the literature.^22,23^ Smith et al reported a case of a 28-year-old patient with perfusion defect on V/Q scan within 4 to 8 hours after cocaine inhalation, and it resolved 4 days later.^22^ In the second case, Ramachandaran et al reported a patient with perfusion defects consistent with high probability for PE on V/Q scan after cocaine inhalation. Pulmonary angiogram was negative, and repeat perfusion scan 2 weeks later was within normal.^23^

## Conclusion

While V/Q scan is a safe screening tool to evaluate for PE, it lacks specificity. Other causes of ventilation or perfusion defects should be considered. In this case, it was secondary to cocaine-induced vasospasm.
